# Etude transversale du statut martial au cours de la grossesse à l'Hôpital Militaire d'Instruction Mohamed V, Rabat

**DOI:** 10.11604/pamj.2023.45.139.37687

**Published:** 2023-07-24

**Authors:** Raoul Karfo, Servilien Mpawenimana, Elie Kabré, Jean Sakandé, Saida Tellal

**Affiliations:** 1 Unité de Formation et de Recherche en Sciences de la Santé, Université Joseph KI-ZERBO, Ouagadougou, Burkina Faso,; 2 Clinique du Laboratoire de Biologie du Centre Médical du Camp General Aboubacar Sangoulé Lamizana, Ouagadougou, Burkina Faso,; 3 Faculté de Médecine et de Pharmacie de Rabat, Rabat, Maroc,; 4 Hôpital Militaire d'Instruction Mohamed V, Rabat, Maroc

**Keywords:** Carence martiale, anémie gravidique, ferritine, fer sérique, hémoglobine, Iron deficiency, anemia during pregnancy, ferritin, serum iron, hemoglobin

## Abstract

La carence en fer est la maladie nutritionnelle très répandue dans le monde. L'anémie au cours de la grossesse est fréquente dans les pays en voie de développement. Notre objectif était de suivre l'évolution du statut martial au cours de la grossesse par l'évaluation des indicateurs biologiques de la carence martiale. Elle était réalisée au service de gynéco-obstétrique à l'Hôpital Militaire d'Instruction Mohamed V (HMIMV) sur 66 patientes que nous avons suivies au cours des trois trimestres de grossesse. Les analyses biochimiques notamment la ferritine, le fer sérique, la protéine C reactive (CRP) et les numérations sanguines ont été effectuées. L'âge moyen était de 28,3 ans avec un écart type de 5,2. La prévalence de la carence martiale a été de 15,2% (n = 10), de 25,8% (n = 17) et de 42,2% (n = 28) respectivement aux premier, deuxième et troisième trimestre. L'anémie gravidique a évolué dans le même sens en passant de 10% (n = 7), 24% (n = 16) à 42% (n = 28). La comparaison des moyennes évolutives des trois trimestres par ANOVA, de la ferritine et du fer sériques, de l'hémoglobine ainsi que de l'hématocrite a montré une différence significative avec p = 0,001. Il existe une forte prévalence de la carence martiale au cours de la grossesse surtout au troisième trimestre qui justifierait la supplémentation systématique en fer.

## Abstract (en)

Iron deficiency is the most widespread nutritional disorder. Anaemia during pregnancy is common in developing countries. The purpose of this study was to check iron levels during pregnancy by evaluating laboratory indicators of iron deficiency. Our study was conducted in the Department of Obstetrics and Gynaecology of the Mohamed V Military Training Hospital (MVMTH) and included 66 patients monitored during three trimesters of pregnancy. Biochemical tests including ferritin, serum iron, CRP and blood counts were performed. The average age of patients was 28.3 years, with a standard deviation of 5.2. The prevalence of iron deficiency was 15.2% (n = 10), 25.8% (n = 17) and 42.2% (n = 28) in the first, second and third trimester, respectively. Anemia progressed from 10% (n = 7), 24% (n = 16) to 42% (n = 28). ANOVA test was used to compare the means for the parameters serum ferritin and iron, hemoglobin and hematocrit within the three trimesters. This showed a significant difference with p = 0.001. This study highlights a high prevalence of iron deficiency during pregnancy, especially in the third trimester, that would justify a systematic iron supplementation.

## Introduction

La carence en fer est la maladie nutritionnelle très répandue dans le monde, touchant non seulement les populations des pays en voie de développement mais aussi celles des pays industrialisés [1]. L'anémie au cours de la grossesse est fréquente dans les pays en voie de développement, où elle touche 30 à 80% des femmes. La carence martiale est la plus responsable de ces anémies. La carence en fer prénatale s'observe chez plus de 30% des personnes enceintes au Canada [2]. Elle est le plus souvent liée à des régimes pauvres en fer [3]. Les conséquences de la carence martiale sont multiples, affectent à la fois la santé maternelle et celle du fœtus en développement. La carence martiale augmente le risque de menaces d'accouchement prématuré, d'hypotrophie du nouveau-né, de mort fœtale intra-utérine [2,4]. Chez la mère, les conséquences sont représentées par les symptômes cardiovasculaires, la baisse de la performance physique et mentale, la réduction de la capacité de résistance aux infections, l'asthénie et la réduction puerpérale du volume sanguin périphérique. Ceci implique dans certains cas un recours à la transfusion sanguine en post – partum [2]. Le but de notre étude est de suivre l'évolution du statut martial au cours de la grossesse par l'évaluation des indicateurs biologiques de la carence martiale.

## Méthodes

**Conception et cadre de l'étude:** il s'agit d'une étude réalisée à l'Hôpital Militaire d'Instruction Mohammed (HMIMV) de Rabat sur 66 femmes enceintes sur une période de onze mois. Sa réalisation a pu bénéficier de l'intervention, l'assistance technique et matérielle de quatre services, en occurrence le service de Gynéco-obstétrique, le laboratoire de Biochimie-Toxicologie, le laboratoire d'Hématologie et enfin le service d'épidémiologie.

**Population de l'étude:** la population ciblée était les femmes enceintes. Les patientes incluses ont été alors suivies, du premier trimestre au troisième trimestre. Ont été incluses dans cette étude, les patientes dont l'âge de la grossesse en cours devait être situé entre huit et quatorze semaines d'aménorrhée (8-14 SA) et ne possédant pas de critères d'exclusion. Toute patiente qui présentait une maladie quelconque essentiellement les cardiopathies, le diabète, l'hypertension artérielle, les hépatopathies, le rhumatisme et les autres pathologies inflammatoires au moment du recrutement n'était pas éligible. Les patientes porteuses de grossesse multiple (gémellaire) ou extra-utérine et les patientes chez qui l'âge gestationnel était au-delà des 14 SA, ont été exclues.

**Analyse en laboratoire:** le dosage de la ferritine était effectué sur le sérum ou le plasma. Il s'agit d'un immunodosage enzymatique fondé sur le principe type « sandwich » réalisé sur le système de chimie clinique Dimension®. Le dosage du fer sérique était fait sur le sérum. Il s'agit d'un dosage par déprotéinisation qui utilise une technique colorimétrique au chromophore FERENE ® en milieu acide (pH 4,5). Le dosage de la CRP était effectué sur le sérum ou le plasma. Le dosage de la CRP ultrasensible sur l'autoanalyseur dimension® RXL était fait par immunoturbidimètrie avec l'utilisation des particules sensibilisées qui sont liées à des anticorps. Les paramètres hématologiques étaient basés sur la numération formule sanguine (hémogramme) à travers laquelle, nous avons apprécié l'évolution des paramètres ciblés. Le principe de base du compteur Coulter® s'appuie sur une mesure par variation d'impédance. Un flux contrôlé du liquide porteur des cellules à compter passe au travers d'un micro-orifice calibré. Le passage d'une cellule modifie le comportement électrique du milieu: l'impédance est fonction de la taille et éventuellement de la nature de la cellule. A ce principe initial de base, on a ajouté, la possibilité de mieux identifier les sous-populations en colorant les cellules puis en mesurant l'absorbance par diffraction de la lumière d'un faisceau laser (Tableau 1). La détermination de la fréquence des patientes ayant développé une carence martiale au cours des trois trimestres a été basée sur les résultats issus du dosage de la ferritine sérique. Un résultat inférieur à 8 ng/ml est qualifié d'hypoferritinémie. Les valeurs diagnostiques de l'anémie gravidique utilisées sont celles recommandées par l'OMS et le *Center of Disease and Control (CDC)* sous formes de gold standard. Ainsi, au premier trimestre l'anémie est définie par un taux d'hémoglobine inférieur à 11g/dl, inférieur à 10,5g/l au deuxième trimestre et inférieur à 11g/dl au troisième trimestre. La carence martiale au stade avancé est définie sur le plan hématologique par la combinaison de trois paramètres hématologiques, dont le taux d'hémoglobine diminué (< 11 g/dl), la microcytose (VGM < 80) et l'hypochromie (TCMH).

**Tableau 1:** paramètres biologiques analysés

	Paramètres	Méthodes de dosage	Réactif	Fabricant	Valeurs physiologiques
Paramètres Biochimiques	Fer sérique	Méthode IRN	Chromophore Ferene	SIEMENS	35-150µg/dl
	Ferritine sérique	Immunodosage en phase hétérogène	Flex™FERR		Femme: 8-252 ng/ml Homme: 26- 388 ng/ml
	CRP sérique	Turbidimétrie Immunodosage Utilisant des particules sensibles (PETIA)	Flex™CRP		0,5-3 mg/l
Paramètres hématologiques	Nombre de globules rouges	Variation de l'impédance	Complexe des reactifs: Solution de lyse, le diluant, PAK	BECKMAN COULTER	Femme: 4,5x10^6^/mm^3^ Homme: 4,5- 5,5x10^6^/mm^3^
	Taux d Hemoglobine	Photométrie			Femme: 12-15 g/dl, Homme: 13-18 g/dl
	VGM	Obtenu par calcul intégré dans l'automate de mesure			80-100 fl
	Hematocrite				Femme: 37-48%, Homme: 40- 52%
	TCMH				28-32pg
	CCMH				33-36 g/dl

**Collecte des données:** les données étaient recueillies suite à un questionnaire dont les réponses étaient automatiquement transcrites sur une fiche d'exploitation préalablement établie. Un consentement éclairé a été d'abord recueilli de la part de chaque patiente avant son inclusion. Les patientes incluses ont été alors suivies, du premier trimestre au troisième trimestre. Au cours de cette étude, les patientes ayant bénéficié d'une supplémentation martiale, sont évaluées au nombre de 35 patientes (soit 53% de l'ensemble de la population étudiée). Néanmoins, ce paramètre n'a pas été exploité du fait que la supplémentation n'a pas été effectuée au même moment (J0) pour toutes les patientes afin de nous permettre de bien établir son impact sur le statut martial.

**Définitions**

**Ferritine:** glycoprotéine renfermant des atomes de fer sous forme ferrique. La ferritine est principalement intracellulaire où elle constitue une forme de réserve échangeable. Son taux sérique est directement corrélé avec les réserves en fer de l'organisme.

**VGM:** c'est la valeur moyenne mesurée du volume de chaque hématie.

**TCMH:** elle correspond à la moyenne du poids d'hémoglobine contenu dans un globule rouge (G.R) et se calcule selon le rapport: Hémoglobine (g/l) / Numération des GR (en millions).

**Analyse statistique:** les données ont été saisies en utilisant le logiciel Microsoft office sous le format Excel 2007 puis les analyses statistiques ont été faites par le logiciel SPSS 17.0. Cette analyse a été subdivisée en deux grandes étapes:

Première étape: les données qualitatives ont été représentées par leurs fréquences et les données quantitatives par leurs moyennes associées à l'écart-type (MAE). Deuxième étape: pour comparer les différents groupes entre eux, nous avons utilisés le test statistique approprié, ainsi que le test de Khi-deux pour la comparaison des pourcentages.

Le test ANOVA (*Analysis Of Variance*) avec mesures répétées a été utilisé pour la comparaison des moyennes des résultats des différents dosages hémato-biochimiques et le choix de la correction par ajustement de BENFERRONI. Les résultats ont été jugés statistiquement significatifs pour un p value < 5%. Le coefficient de corrélation de Pearson a été utilisé pour déterminer la relation entre le niveau des réserves en fer et les paramètres hématologiques.

**Considérations éthiques:** une base de données anonyme a été constituée à partir des dossiers médicaux et biologiques des patients inclus dans l'étude. Aucune information ne permettait d'identifier les patients inclus dans cette étude. La base de données reste une propriété du Service de gynécologie de l'HMIMV, du Service du Laboratoire de Biochimie de l'HMIMV et du service du laboratoire d'hématologie de l'HMIMV. L'étude a été autorisée par les chefs des services impliqués.

## Résultats

### Caractéristiques générales de la population étudiée

Au départ, 82 femmes enceintes ont été incluses. Durant l'étude, 16 patientes étaient perdues de vue. Après élimination des résultats incomplets, le taux de participation est de 80,5%, soit 66 patientes. La majorité de nos patientes était d'âge compris entre vingt et quarante ans. L'âge moyen était de 28,3 ans avec un écart type de 5,2. Parmi les patientes incluses, certaines sont primigestes d'autres multigestes (gestitité supérieure ou égale à deux). Le nombre de fausses couches antérieures à la grossesse en cours était de 0,2 fausses couches par patiente en moyenne. L'espace intergénésique moyen est de 57 mois (avec une médiane m = 93 mois).

**Principaux résultats:** l'évolution des principaux paramètres biochimiques et hématologiques (ferritine sérique, fer sérique, globules rouges, hématocrite) a suivi une allure de décroissance linéaire, du premier au troisième trimestre avec une différence sur les moyennes très statistiquement significative. On note une différence non significative entre la moyenne de l'hémoglobine érythrocytaire au deuxième trimestre par rapport à celles du premier et troisième trimestre. La population étudiée a été repartie selon les résultats des paramètres biologiques dans le Tableau 2. La fréquence des patientes présentant une carence martiale a évolué de 15,2% (n=10), de 25,8% (n=17) et de 42,2% (n=28) respectivement au premier, deuxième et troisième trimestre. La corrélation de Pearson est significative entre la ferritine sérique et l'hématocrite au deuxième et troisième trimestre. Elle est aussi significative entre le niveau des réserves en fer et la CCMH au troisième trimestre (Tableau 3).

**Tableau 2:** répartition de la population étudiée selon les résultats des paramètres biologiques

Variables	Premier trimestre		Deuxième trimestre		Troisième trimestre	
	**N**	**%**	**N**	**%**	**N**	**%**
**Ferritine (ng/ml)**						
8-252	56	84,8	49	74,2	36	58,6
< 8	10	15,2	17	25,8	28	42,2
**Fer (µg/L)**						
35-150	64	99,7	59	90	18	27
<35	2	0,3	7	10	48	73
**Hb(g/dl)**						
>10,5 et 11	59	90	10	15	28	42
<10,5 et 11	7	10	56	75	38	58
VGM (fl)						
80-100	58	88	57	86	53	80
<80	8	12	9	14	13	20
**Hématocrite (%)**						
>33	59	89	49	74	49	59
<33	7	11	17	26	27	41
**TCMH (pg)**						
27-33	58	88	57	87	53	81
<27	8	12	9	13	13	19
**CCMH (g/dl)**						
32-36	60	91	62	94	60	91
<32	6	6	4	6	6	9
**RDW (%)**						
≤15	57	86	59	89	48	73
>15	9	14	7	11	18	27

**Tableau 3:** corrélation entre l'état des réserves en fer et l'anémie ferriprive

Variables		r	p
**Premier trimestre**			
**Ferritine**	Hémoglobine	0,13	0,26
	Hématocrite	0,1	0,41
	Volume globulaire moyen (VGM)	0,19	0,11
	Teneur corpusculaire moyenne en hémoglobine (TCMH)	0,13	0,21
	Concentration corpusculaire moyenne en hémoglobine (CCMH)	0,09	0,43
**Deuxième trimestre**			
Ferritine	Hémoglobine	0,26	0,83
	Hématocrite	0,35*	0,003
	Volume globulaire moyen (VGM)	0,19	0,11
	Teneur corpusculaire moyenne en hémoglobine (TCMH)	0,19	0,11
	Concentration corpusculaire moyenne en hémoglobine (CCMH)	0,06	0,61
**Troisième trimestre**			
Ferritine	Hémoglobine	0,04	0,75
	Hématocrite	0,25	0,05
	Volume globulaire moyen (VGM)	0,16	0,21
	Teneur corpusculaire moyenne en h émoglobine (TCMH)	0,09	0,49
	Concentration corpusculaire moyenne en hémoglobine (CCMH)	0,3	0,01

r: cœfficient de corrélation de Pearson

L'anémie gravidique a évolué de 10% (n = 7), 24% (n = 16) à 42% (n = 28) respectivement au premier, deuxième et troisième trimestre. Sur les 66 naissances qui étaient attendues, deux cas sur 37 naissances ayant eu lieu à l'HMIMV, ont été caractérisés par un accouchement prématuré et par une mort fœtale intra-utérine (MFIU). Les résultats moyens des bilans martiaux anténatals de la mère de l'enfant prématuré ont montré une microcytose très profonde (VGM 15%) avec un effondrement des réserves martiales (une hypoferritinémie très prononcée).

## Discussion

**Figure 1:**
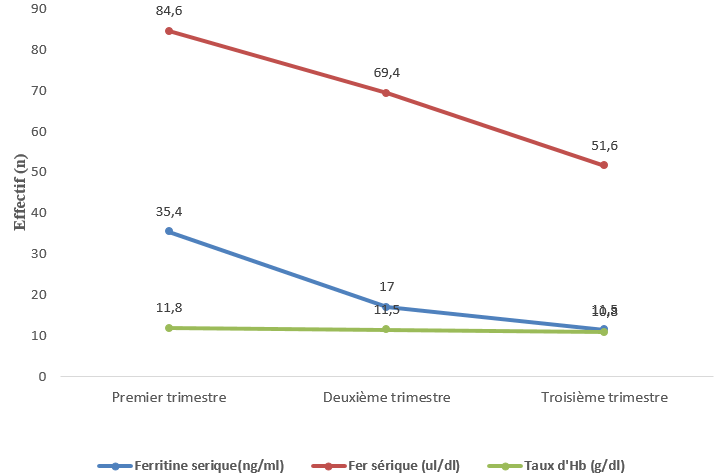
évolution moyenne des paramètres hémato-biochimiques au cours des trois trimestres

Notre étude avait pour objectif de suivre l'évolution du statut martial au cours de la grossesse par l'évaluation des indicateurs biologiques de la carence martiale. La prévalence de la carence martiale a été de 15,2% (n=10), de 25,8% (n = 17) et de 42,2% (n=28) respectivement aux premier, deuxième et troisième trimestre. L'anémie gravidique a évolué dans le même sens en passant de 10% (n=7), 24% (n=16) à 42% (n= 28). Les principaux paramètres biochimiques et hématologiques ont suivi une décroissance, du premier au troisième trimestre avec une différence sur les moyennes statistiquement significative (Figure 1). Cette régression concerne en premier lieu le fer des réserves et le fer sérique, et en seconde position le taux de l'hémoglobine érythrocytaire dont la régression n'a pas été très marquée comme celle des deux premiers paramètres. Enfin, le dernier stade correspond à l'anémie ferriprive où la chute du taux d'hémoglobine en dessous du seuil limite fait reconnaître l'anémie [5,6]. La différence sur les moyennes des concentrations sériques de paramètres du bilan martial et de l'hémoglobine érythrocytaire aux différents trimestres s'explique par l'augmentation des besoins spécifiques pour la croissance fœtale [7]. Le stock du fer augmente de 3mg/j pour atteindre 75 mg/kg chez le nouveau-né à terme [8].

Dans notre étude, la différence significative des moyennes d'hémoglobine érythrocytaire au premier trimestre et au troisième trimestre pourrait être interprétée par le phénomène d'hémodilution qui est généralement accentué à partir de la 35^e^semaine d'aménorrhée [9]. Le concept de la carence martiale sans anémie n'est pas nouveau car l'hématologue américain E. Beutler a pu démontrer dans une étude en double aveugle et contrôlée contre placebo que les femmes enceintes présentant une fatigue comme symptôme majeur d'une carence martiale modérée sans anémie préféraient du comprimé du fer aux placebos [10]. Dans la présente étude, la présence des patientes au premier trimestre avec déjà une hypoferritinémie, témoigne que certaines débutent leur grossesse avec une pré-carence martiale comme c'est décrit dans la littérature [11]. Cette situation est très fréquente dans les pays en voie de développement où l'on trouve un nombre croissant de femmes en âge de procréer avec une balance en fer qui est déficitaire [12]. La carence martiale en milieux ruraux marocains est due à une insuffisance d'apport alimentaire en fer et à sa biodisponibilité limitée dans les repas [13,14].

Une étude réalisée en Côte d'Ivoire a montré que 2/3 des patientes avaient une anémie ferriprive (66%) et elles présentaient un véritable déficit en fer à l'égard de leurs VGM et CCMH diminués de façon remarquable [6]. Des travaux réalisés en France ont pu relever que 60 à 80% des femmes enceintes en fin de grossesse présentaient des valeurs anormales pour les principaux marqueurs d'évaluation du statut en fer et ces déficiences sont suffisamment intenses pour être responsables d'une anémie ferriprive chez 10 à 30% des femmes enceintes françaises [15]. Naima Belkadi *et al*. lors d'une étude sur l'anémie ferriprive au cours de la grossesse dans la wilaya de Blida au Nord de l'Algérie ont trouvé que la prévalence de la carence martiale chez les femmes enceintes de neuf mois était de 46% [16]. L'anémie ferriprive constitue un facteur de risque d'accouchement prématuré qui a pour corollaire un petit poids à la naissance et possiblement une mauvaise santé néonatale [17-19]. La prévalence de faible poids à la naissance (FPN) est plus élevée dans les pays en voie de développement que dans les pays développés. En Inde, dans les milieux pauvres, la prévalence est de 39,1%. Dans les milieux urbains du Bangladesh, elle est de 36,8%. Dans certaines localités des pays d'Asie, elle est de 19%, tandis que cette prévalence est faible dans la ville de Babul au Nord de l'Iran (7,7%) [20]. Nous avons constaté un seul cas de FPN sur les 37 naissances issues de parturientes faisant partie de l'étude. Un seul accouchement prématuré a été aussi remarqué. Dans ce dernier cas, il y a une grande présomption que la carence martiale soit à l'origine.

En effet, les résultats moyens des bilans martiaux anténatals de la mère ont montré une microcytose très profonde (VGM 15%) avec un effondrement des réserves martiales (une hypoferritinémie très prononcée). Par ailleurs, la patiente nous a confié qu'elle consommait beaucoup de thé ce qui nous porte à croire que la carence martiale en serait parmi les causes de la naissance prématurée. Parmi les autres conséquences de la carence martiale, plusieurs études réalisées s'accordent en désignant la déficience en fer comme responsable de l'augmentation des mort-nés et de la morbi-mortalité maternelle pendant, avant ou après l'accouchement [21,22]. Un cas de mort-né (par mort fœtale intra-utérine MFIU) a été remarqué au cours de cette étude. Toutefois, il nous est difficile d'affirmer la relation de causalité en rapport avec la carence martiale, étant donné que les résultats biologiques de la patiente étaient autour de la limite normale. Néanmoins, on remarque un VGM normal, un taux d'hémoglobine diminué avec diminution du pourcentage de l'hématocrite. Ces résultats sont en faveur d'une hémodilution.

Les limites de cette étude sont que la supplémentation et la prise du fer de certains compléments multivitaminés n'étaient pas prises en considération lors de l'exploitation des données. La prévalence de l'anémie ferriprive n'a pas été explicitement explorée en raison du coefficient de saturation de la transferrine que nous n'avons pas eu l'occasion de déterminer. Cette étude ouvre la perspective d'une grande étude multicentrique couvrant la majorité des centres de santé, de la ville de Rabat et de ses environs.

## Conclusion

Dans notre étude certaines patientes ont eu un statut martial en dessous de la limite normale au premier trimestre, d'autres au deuxième avant que leur fréquence soit augmentée au troisième trimestre. Bien que nous n'ayons pas trouvé des facteurs de risque de la carence martiale dans la population étudiée, la supplémentation basée sur l'apport du fer et des folates doit être mise en route le plutôt que possible, chez les patientes présentant un risque potentiel de développer la carence martiale précocement.

### Etat des connaissances sur le sujet

- *L'anémie au cours de la grossesse est fréquente au Maroc, au Burkina Faso;*
- *Les conséquences de la carence martiale sur la santé maternelle;*
- *Les conséquences de la carence martiale sur le fœtus en développement*.

### Contribution de notre étude à la connaissance

- *Meilleure interprétation des marqueurs du statut martial au cours de la grossesse;*
- *Fournit des informations sur l'évolution du statut martial au cours de la grossesse;*
- *Intérêt du démarrage précoce de la supplémentation basée sur l'apport du fer et des folates chez les patientes présentant un risque potentiel de développer la carence martiale précocement*.
